# Cervical Cancer Prevention in El Salvador: Gains to Date and Challenges for the Future

**DOI:** 10.3390/cancers14112776

**Published:** 2022-06-03

**Authors:** Karla Alfaro, Montserrat Soler, Mauricio Maza, Mauricio Flores, Leticia López, Juan C. Rauda, Andrea Chacón, Patricia Erazo, Nora Villatoro, Eveline Mumenthaler, Rachel Masch, Gabriel Conzuelo, Juan C. Felix, Miriam Cremer

**Affiliations:** 1 Basic Health International, Pittsburgh, PA 15205, USA; kalfaro@basichealth.org (K.A.); llopez@basichealth.org (L.L.); jcrauda@basichealth.org (J.C.R.); emumenthaler@basichealth.org (E.M.); rmasch@basichealth.org (R.M.); gconzuelo@basichealth.org (G.C.); mcremer@basichealth.org (M.C.); 2 Ob/Gyn and Women’s Health Institute, Cleveland Clinic, Cleveland, OH 44195, USA; 3 Cancer Program, Pan American Health Organization, Washington, DC 20037, USA; mazamau@paho.org; 4 Comité Asesor de Prácticas en Inmunizaciones de El Salvador, San Salvador C.P. 01101, El Salvador; mernesto.flores@salud.gob.sv; 5 Unidad de Cáncer, Ministerio de Salud de El Salvador, San Salvador C.P. 01101, El Salvador; marina.chacon@salud.gob.sv (A.C.); mayra.erazo@salud.gob.sv (P.E.); nora.villatoro@salud.gob.sv (N.V.); 6 Department of Pathology, Medical College of Wisconsin, Milwaukee, WI 53226, USA; jcfelix@mcw.edu

**Keywords:** El Salvador, cervical cancer, cervical pre-cancer, human papillomavirus (HPV), screen-and-treat

## Abstract

**Simple Summary:**

Cervical cancer is a leading cause of death for women in low-resource areas around the world. However, the disease can be prevented through vaccination, screening, and early treatment. Recently, the World Health Organization released a strategy with the goal of eliminating cervical cancer through a combination of these three preventive strategies. In El Salvador, a screening program has been in place for some years, and vaccination is in its early stages. Here, we describe the Salvadoran experience and discuss successes to date and challenges for the future. We also provide recommendations that may be helpful for other countries working to meet the WHO elimination goals.

**Abstract:**

Cervical cancer is preventable through vaccination, early detection, and the treatment of pre-cancerous lesions. However, global inequalities mean that the disease remains a leading cause of cancer death around the world, with over 80% of new cases and 90% of deaths occurring in low- and middle-income countries (LMICs). In El Salvador, joint efforts between the Ministry of Health (MoH) and the non-profit organization Basic Health International (BHI) have been in place since 2008, with the goal of reducing the country’s disease burden. While the World Health Organization’s (WHO) call to action to eliminate cervical cancer provided worldwide momentum to implement new public health initiatives, the COVID-19 pandemic disrupted ongoing programs and jeopardized plans for the future. The purpose of this manuscript is to describe the progress that El Salvador has achieved in improving cervical cancer prevention, the impact of the pandemic on current strategies, and potential solutions that can help the country meet the WHO’s strategic targets by 2030 to accelerate the elimination of cervical cancer.

## 1. Background

Cervical cancer is a disease of inequality. Wealthy countries have access to preventive strategies that have significantly reduced incidence and mortality rates in the last 50 years, but low- and middle-income countries (LMICs) continue to bear a large burden of disease [1]. It is estimated that 80% of new cases and 90% of deaths from cervical cancer occur in LMICs, and these numbers are expected to grow in the next decade [2]. In light of these considerations, the World Health Organization (WHO) released a call to action in 2018 to eliminate cervical cancer as a public health problem [3]. The WHO strategy sets three global targets to be reached by 2030 in order to accelerate elimination: (1) vaccinating 90% of girls by age 15 with the human papillomavirus (HPV) vaccine, the causal agent of the disease, (2) screen 70% of women at age 35, and again at age 45, with a high-performance test, and (3) treat 90% of women with underlying cervical disease [4]. Meeting these goals would save 300,000 lives by 2030, 13.4 million lives by 2070, and 62 million lives by 2120 [5].

While the WHO’s strategy presents challenges to resource-poor countries with competing health priorities, the call to action has provided momentum to increase access to cervical cancer-preventive services. El Salvador is well positioned to demonstrate an effective implementation of cervical cancer control programs. The country has historically high rates of incidence and mortality (13.1 and 7.43 in 100,000, age-adjusted, respectively) [1]. The sociocultural context complicates access to cancer prevention services. Almost 40% of the population lives in rural areas, and gang-related violence, poverty, and out-migration remain pervasive problems. The Ministry of Health operates a network of municipal clinics that offer free-of-cost primary care services, in addition to regional hospitals in urban areas that offer more specialized care. For many women, transportation to clinics and hospitals is a significant hurdle.

Since 2008, a collaboration between the Ministry of Health (MoH) and Basic Health International (BHI), a non-profit organization, has resulted in a range of initiatives to strengthen cervical cancer prevention in the country [6]. Previously, the national screening program relied on colposcopy and/or cytology (Pap test), which involved multiple visits for each patient and resulted in a very low national coverage (estimates ranged from 19% [7] to 44% [8]). In 2012, a partnership between the MoH and BHI resulted in a donation of low-cost HPV tests (careHPV, Qiagen, Gaithersburg, MD, USA) to the country. The tests were utilized to conduct a demonstration project that eventually reached 28,000 women. In the first stage, 2000 women were assigned to either screen-and-treat or colposcopy management [9]. Screen-and-treat approaches, where all screen-positive women are offered treatment without a biopsy-confirmed diagnosis, are recommended by the WHO in low-resource settings [10]. In El Salvador, the initial pilot was gradually expanded to an additional 8000 women. Outcomes revealed differences in loss to follow-up, with 88.3% of HPV-positive women in the screen-and-treat group completing treatment in primary care clinics in 6 months, compared to 44.2% of HPV-positive women who were referred to colposcopy management within the same time period [11].

Following the success of this project, the screen-and-treat program was scaled-up in the paracentral region [12]. Cost-effectiveness analyses demonstrated the long-term advantages of this approach in the Salvadoran context [13,14]. Findings were presented to public health officials, and resulted in the incorporation of HPV testing into the national cervical cancer control guidelines [15], making El Salvador one of the first countries to do so in the region. In 2020, the COVID-19 pandemic disrupted health systems worldwide, but secondary prevention services continued as local regulations allowed. The purpose of this manuscript is to describe the progress of cervical cancer prevention programs in El Salvador, review the challenges presented by the COVID-19 pandemic, and outline potential future directions for the country.

## 2. The HPV Screening Program in El Salvador

**Figure 1:**
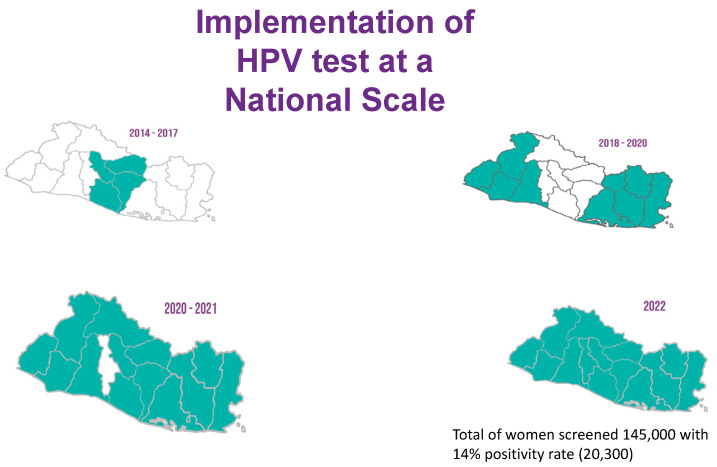
National scale-up of the primary HPV screen-and-treat program in El Salvador.

In 2017, after the initial success of a primary HPV-based screen-and-treat demonstration project, El Salvador was able to obtain grant funding for further HPV tests in addition to HPV vaccines. The MoH included the procurement of HPV tests in its 2022 annual budget, cementing the screen-and-treat program as part of the national cervical cancer control effort. Today, HPV screening is available across the network of primary care units distributed in all five regions of the country (Figure 1). Provider training for implementation in the fifth and final region, which includes the metropolitan area of San Salvador, began in March 2022. Since its inception, the program was designed to rely on the existing public health infrastructure. As technical advisors, BHI has followed a “train-the-trainer” approach with MoH personnel to ensure long-term sustainability. 

Multi-disciplinary health teams form the backbone of the HPV screening program. These consist of healthcare providers at various levels, including general practitioners who head municipal health clinics, nurses at the clinics, educators and community health promoters, among others. Health promoters are the first line of advocacy and education. They are responsible for contacting women due for screening to set up appointments at local clinics according to their catchment area. Currently, El Salvador’s guidelines recommend HPV testing for women aged 30–59, and cytology for those between 20 and 29 and over 59 years of age. Participants of the HPV program receive an educational session taught by nurses on arrival at the local clinic. Women then undergo a provider sample collection using careHPV, which detects 14 types of high-risk HPV (pooled, not genotyped). Screened women are given an appointment to return after 4 weeks for their test results. HPV positive women must schedule the second appointment at the nearest treatment center. There are 74 such centers strategically distributed throughout the country. On the second visit, those who test positive are offered a pelvic exam to visually assess eligibility for the ablation treatment and, if eligible, gas-based cryotherapy. HPV testing, a visual assessment, and treatment are performed by trained general practitioners or gynecologists. Women who are deemed ineligible for treatment during the visual assessment are referred to colposcopy and biopsy by colposcopists at regional hospitals. Criteria for ablation treatment ineligibility are drawn from WHO guidelines, and include a lesion that is larger than 75% of the cervix, enters the endocervical canal, or is suspicious for cancer, or cases where the squamocolumnar junction is not fully visible.

**Figure 2:**
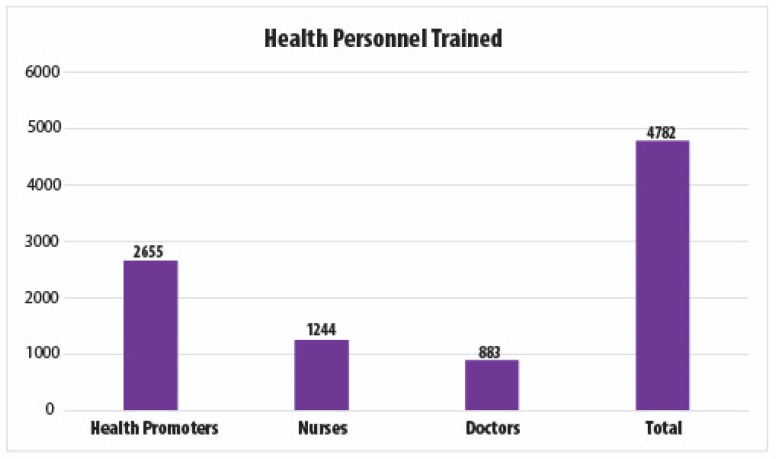
ECO team members trained in El Salvador as part of the national HPV screening program.

To date, over 4500 healthcare providers have been trained as part of the screen-and-treat program (Figure 2) and 145,000 women have been screened. The HPV positivity rate is approximately 14%. Women who test negative return for re-testing after five years. Those who test positive return at one year after the ablation treatment, and if they test positive again, they are referred to colposcopy and biopsy. Although we know that approximately 88% of HPV-screened women have received cryotherapy treatment to date, the data on treatment refusals, adherence to colposcopy and biopsy, or treatment follow-up are not currently available. While El Salvador has added outcomes of its cervical cancer prevention programs to its epidemiological surveillance system since 2012, data entry across hospitals is not consistent. Efforts by the MoH are underway to improve surveillance, including the training of data entry specialists.

After the WHO call for elimination was released, the MoH engaged in further efforts to maximize the impact of this opportunity in El Salvador, with two important outcomes. First, the MoH released a new screening target to increase the number of eligible women screened to 15% per year for the next 5 years, which would achieve the WHO target of 70% of eligible women screened by 2030. Second, after a series of stakeholder meetings that included MoH and BHI personnel, changes were planned to the national cervical cancer guidelines. BHI had successfully completed several projects on the feasibility of HPV self-collection in hard-to-reach areas [16,17,18]. Self-collection allows women to collect their own sample using a small brush, circumventing the need for a speculum exam. BHI also participated in the review of WHO guidelines for thermal ablation [19], an alternative treatment to cryotherapy that does not require a consumable (i.e., compressed gas) and can be applied with a portable, battery-operated device. The BHI team introduced thermal ablation to El Salvador through two ongoing research protocols in collaboration with the MoH and the Salvadoran Social Security Institute. (NCT03084081 and NCT03429582). Both HPV self-sampling and thermal ablation are expected to be endorsed in guidelines to be released in 2022. These innovations have the potential to increase access to screening and treatment, particularly among rural and hard-to-reach populations. 

## 3. HPV Vaccination in El Salvador

The MoH introduced HPV vaccination in 2020. The WHO call to action coincided with a donation from the World Bank, providing the necessary momentum to begin implementation efforts. An advisory committee was convened, and the MoH settled on a school-based strategy targeting 9-year-old girls for a first dose, with a second dose administered after six months. Quadrivalent Gardasil (Merck & Co., Kenilworth, NJ, USA) was the vaccine of choice. Usually, childhood vaccines are given at local health units, but given the need for two doses and the target ages, it was determined that a school-based approach would result in better follow-up. HPV vaccination was projected to start in April 2020 during the Americas Vaccination Week, with the goal of vaccinating approximately 55,000 girls the first year. The HPV vaccine was also included in El Salvador’s vaccination schedule. Finally, the vaccine data collection system was prepared to receive general outcomes of the HPV vaccination effort. As the program was about to begin, COVID-19 was declared a pandemic by the WHO and became a national health priority. The HPV screening and vaccination programs were immediately suspended. 

## 4. The Impact of COVID-19

In March 2020, as COVID-19 cases began to rise nationally, public health services focused on containing the pandemic. Women who had been screened as part of the HPV screen-and-treat program had follow-up care delayed for several months. The planned April start date for the new HPV vaccination campaign was re-scheduled to November 2020. As non-essential medical services slowly restarted in late 2020, an urgent COVID-19 vaccination campaign was prioritized. Screening and vaccination initiatives for cervical cancer gradually resumed.

The true impact of the pandemic on routine medical services has only recently begun to surface. In the Occidental and Oriental regions of El Salvador, an estimated 5000 HPV-positive women were awaiting follow-up care since the beginning of the lockdowns in 2020. Thus, in collaboration with BHI, a funding opportunity was identified, and a project initiated with the goal of locating these women and providing follow-up care. Since MoH providers and laboratories remained at capacity with COVID-related care, private colposcopists and pathology laboratories were engaged to conduct this intervention. From March 2021 to March 2022, 3017/5000 (60%) screen-positive women were identified, and efforts are ongoing to locate the remaining 1983/5000 (40%). Of those accounted for, 702/3017 (23%) had already received follow-up care at private clinics, 210/3017 (7%) refused further care, and 96/3017 (3%) were lost to follow-up (had moved, migrated out of the country, died, etc.). The remaining 2009/3017 (66.5%) underwent colposcopy and biopsy as part of this project. Laboratory analyses have been completed for 1990/2009 (99%). Results showed high rates of high-grade pre-cancer at 23% (452/1990) and invasive cancer at 1.3% (26/1990). Although there were no comparable pre-pandemic figures available, findings from the original HPV screen-and-treat demonstration project provided a frame of reference. Among HPV-positive women in the colposcopy management group who completed biopsy within 6 months, 56/385 (15%) were diagnosed with high-grade pre-cancer and 1 (<1%) with invasive cancer [11]. These women were similarly recruited from a semi-rural screening population. Thus, rapid action will be necessary to reduce backlogs and achieve elimination targets. 

The HPV vaccination effort has also faced challenges. Since public schools were shut down for most of 2020 and part of 2021, the MoH decided to shift its strategy to an at-home approach. The age range was also expanded to encompass girls aged 9 to 11 years old. Despite these adjustments, the total number of first doses provided was 15,286, 28% of the original target for 2020. Although these figures fell short of initial projections, the inclusion of the HPV vaccine in the national budget means that the procurement of HPV doses in the next few years is assured. This, in turn, should facilitate meeting elimination goals.

## 5. Challenges for the Future

El Salvador achieved significant gains over the last decade to reduce its cervical cancer burden. Since 2022, there is a scalable screening program in all regions of the country and HPV test kits were purchased solely with government funds. The WHO call for elimination provided additional motivation to set defined screening targets and begin the implementation of a new HPV vaccination program, despite the challenges of the pandemic. However, significant barriers remain, many of which represent broader issues that can impede elimination goals globally. Although HPV screening is highly acceptable to patients and providers, there are limited low-cost options. In addition, many existing HPV tests require complex equipment or long processing times. Inexpensive, rapid HPV tests are in development, but none are yet available that allow for the maintenance of a nation-wide, single-day screen-and-treat program. There is also an urgent need for affordable and accessible triage options that can minimize over-treatment and more efficiently channel resources. Better follow-up options for women ineligible for ablation, who may be at greater risk for invasive cancer, are also lacking. Innovative and affordable point-of-care screening and treatment alternatives will be necessary to meet elimination goals.

In terms of primary prevention, the HPV vaccination program in El Salvador is still in early adoption. It is a positive sign that the vaccine has been included in the national vaccination schedule, which will greatly assist in ensuring its sustainability. However, original targets were not reached and it will be important to devise strategies to reach those populations. As in other countries, even if vaccines become widely available, effective interventions to overcome vaccine hesitancy will be essential. On the other hand, if a single-dose schedule is eventually introduced, this would facilitate implementation by reducing costs and avoiding loss to follow-up. 

The COVID-19 pandemic has also highlighted the need to strengthen health systems globally, with cervical cancer programs in particular. In El Salvador, the return of screening has been gradual and irregular due to recurring infection waves, and there is a backlog of women waiting for results or treatment. While other health emergencies will always exist, there are strategies that can help mitigate these challenges. For example, HPV self-collection can allow for at-home testing and including HPV vaccination in national schedules can help prioritize cervical cancer prevention. 

## 6. Conclusions 

In order for LMICs to meet the WHO’s elimination goals by 2030, more affordable and accessible vaccination and screening modalities are necessary, but are not currently sufficient. It is also essential to follow evidence-based strategies and recommendations to implement and sustain population-level programs. Our experiences in El Salvador have taught us valuable lessons in this regard. First and foremost, it is crucial to engage stakeholders at the early planning stages of any project. At the local level, a joint BHI and MoH team organized meetings and workshops with MoH staff at various levels, professional organizations, and other actors working in the cervical cancer space in order to build interest in a pilot demonstration. We also activated an international network of academic connections, which proved valuable in bringing expertise to the design and implementation of the project. The MoH has been extremely receptive of these contributions, and this has helped to build an in-country capacity across multiple sectors. We would encourage any government team involved in screening or vaccination initiatives to seek out partnerships with research or non-profit groups that can bring flexible skills and resources to the development of a new initiative. 

Once a program is ready for implementation, it is crucial to design an education and awareness plan, not only at the community level, but also targeting the staff that will run it on a day-to-day basis. In El Salvador, there are training and refresher courses for clinicians and health promoters, which ensure that providers at all levels are knowledgeable and motivated to recruit participants. On the more practical side, negotiating with local distributors to supply tests and necessary materials can be helpful in avoiding customs delays and cutting down costs. Finally, surveillance and data collection, at any scale that is feasible, are absolutely necessary to gauge program success and detect areas where more work is needed. In our case, data were also essential to effect guideline change and ensure a meaningful and sustainable impact. The WHO call for elimination has placed cervical cancer prevention at the front and center of global health. It is crucial that governments, funding agencies, researchers, and other stakeholders take advantage of the current moment to meet elimination targets and ensure that future generations are free of this preventable disease.

## Data Availability

The data shown is aggregated data from the Ministry of Health and not publicly available.
